# Should Patients with Kearns-Sayre Syndrome and Corneal Endothelial Failure Be Genotyped for a *TCF4* Trinucleotide Repeat, Commonly Associated with Fuchs Endothelial Corneal Dystrophy?

**DOI:** 10.3390/genes12121918

**Published:** 2021-11-29

**Authors:** Lubica Dudakova, Pavlina Skalicka, Alice E. Davidson, Amanda N. Sadan, Monika Chylova, Helena Jahnova, Nicole Anteneova, Marketa Tesarova, Tomas Honzik, Petra Liskova

**Affiliations:** 1 Department of Paediatrics and Inherited Metabolic Disorders, First Faculty of Medicine, Charles University and General University Hospital in Prague, 128 08 Prague, Czech Republic; lubica.dudakova@lf1.cuni.cz (L.D.); pavlina.skalicka@vfn.cz (P.S.); monika.chylova@lf1.cuni.cz (M.C.); helena.jahnova@vfn.cz (H.J.); nicole.anteneova@vfn.cz (N.A.); marketa.tesarova@lf1.cuni.cz (M.T.); tomas.honzik@vfn.cz (T.H.); 2 Department of Ophthalmology, First Faculty of Medicine, Charles University and General University Hospital in Prague, 128 08 Prague, Czech Republic; 3 UCL Institute of Ophthalmology, London EC1V 9EL, UK; alice.davidson@ucl.ac.uk (A.E.D.); amanda.sadan.18@ucl.ac.uk (A.N.S.); 4 Moorfields Eye Hospital, London EC1V 2PD, UK

**Keywords:** Kearns-Sayre syndrome, corneal endothelium, CTG18.1, *TCF4*, corneal dystrophy, endothelial failure, exome sequencing

## Abstract

The aim of this study was to describe the ocular phenotype in a case with Kearns-Sayre syndrome (KSS) spectrum and to determine if corneal endothelial cell dysfunction could be attributed to other known distinct genetic causes. Herein, genomic DNA was extracted from blood and exome sequencing was performed. Non-coding gene regions implicated in corneal endothelial dystrophies were screened by Sanger sequencing. In addition, a repeat expansion situated within an intron of *TCF4* (termed CTG18.1) was genotyped using the short tandem repeat assay. The diagnosis of KSS spectrum was based on the presence of ptosis, chronic progressive external ophthalmoplegia, pigmentary retinopathy, hearing loss, and muscle weakness, which were further supported by the detection of ~6.5 kb mtDNA deletion. At the age of 33 years, the proband’s best corrected visual acuity was reduced to 0.04 in the right eye and 0.2 in the left eye. Rare ocular findings included marked corneal oedema with central corneal thickness of 824 and 844 µm in the right and left eye, respectively. No pathogenic variants in the genes, which are associated with corneal endothelial dystrophies, were identified. Furthermore, the CTG18.1 genotype was 12/33, which exceeds a previously determined critical threshold for toxic RNA foci appearance in corneal endothelial cells.

## 1. Introduction

Kearns-Sayre syndrome (KSS) spectrum is a rare disorder caused by single, large-scale mitochondrial DNA (mtDNA) deletions. This spectrum is characterized by a multisystem involvement including ptosis and/or ophthalmoplegia and at least one of the following signs including ataxia, pigmentary retinopathy, cardiac conduction defects, hearing impairment, failure to thrive, short stature, cognitive involvement, tremor, and cardiomyopathy [1,2].

An additional rarely observed ocular feature includes corneal involvement [3,4]. KSS spectrum is documented with at least eight cases involving decreased corneal endothelial cell density, pleomorphism, and polymegathism. Occasionally, corneal oedema or excrescences resemble the common disorder Fuchs endothelial corneal dystrophy (FECD) [4,5,6]. In addition, bilateral primary corneal endothelial failure manifesting in adulthood can be present in posterior polymorphous corneal dystrophy (PPCD) [7]. Both the FECD and PPCD are genetically heterogenous. However, the most common cause of FECD is the expansion of a non-coding repeat element (termed CTG18.1), which is present in up to 80% of patients with FECD [8].

Congenital cataracts have not been observed, specifically in relation to KSS. However, they have been described in one case as the first symptom of a neuromuscular disease caused by a novel single large-scale mitochondrial DNA deletion [9].

Although corneal endothelial dysfunction in patients with KSS has been attributed to mitochondriopathy [3], the possible involvement of additional genetic causes has not been previously studied.

In this article, we report ocular findings and genomic DNA analysis in a case diagnosed with KSS, as well as bilateral corneal endothelial failure and paediatric cataracts.

## 2. Materials and Methods

The proband, which is known to carry a single ~6.5 kB mtDNA deletion in blood, buccal smear, and urinary epithelial cells [10], underwent a general systemic evaluation and detailed ophthalmic examination. This included measurements of the best corrected visual acuity (BCVA) extrapolated to decimal values using Snellen charts, noncontact specular microscopy (Noncon ROBO Pachy SP-9000, Konan Medical Inc., Tokyo, Japan), static perimetry (M700, Medmont International, Nunawading, Australia), and spectral domain optical coherence tomography (SD-OCT; Spectralis, Heidelberg Engineering GmbH, Heidelberg, Germany).

Genomic DNA was extracted from venous blood (Gentra Puregene Blood Kit, Qiagen, Hilden, Germany), according to the manufacturer’s instructions. Exome sequencing was performed using a SureSelect Human All Exome V6 capture kit (Agilent, Santa Clara, CA, USA). Generated libraries were sequenced on a NovaSeq 6000 sequencer (Illumina, San Diego, CA, USA). Bioinformatical data analysis was performed as previously described [11]. Rare variants with minor allele frequency ≤0.005 as per gnomAD v2.1.1 [12] in genes, which are associated with corneal dystrophies (PanelApp, version 1.6) and congenital cataracts (PanelApp, version 2.84) were filtered [13]. Conventional Sanger sequencing was used to screen *OVOL2* and *GRHL2* regulatory regions, which was previously implicated in the pathogenesis of PPCD type 1 and 4, respectively [14,15].

Finally, we determined the CTG18.1 repeat length in *TCF4* implicated in the development of ~80% of cases with FECD using the short tandem repeat (STR) assay, as previously described [8,16].

## 3. Results

The patient had no family history of a rare disorder that manifested in childhood or adolescence. His mother died at the age of 45 due to breast cancer, while his 72-year-old father and 37-year-old brother were healthy at the time of the last follow-up. In addition, his mother reportedly had a cataract surgery performed at the age of 35 years. However, no further details are known.

The proband was diagnosed with bilateral cataracts before the age of 6 years and underwent surgery in the left eye at the age of 10 years. A secondary artificial lens was implantation 7 years later. The right eye was similarly affected, but did not have cataract surgery. The patient noticed a gradual development of the bilateral ptosis during the 15 years since the surgery. Progressive sensorineural hearing loss started at the age of 14. In addition, corneal dystrophy was documented at the age of 25 years. A distal weakness of the limbs manifested at 27 years, together with the slowly progressing muscle atrophy. Electromyography findings supported motoric axonopathy. A neurological examination confirmed the atrophy of *m. temporalis* and *m. masseter*, mild atrophy of distal muscles in the upper extremities with hyporeflexia, and more pronounced atrophy in lower extremities with areflexia. Repeated cardiological examinations including echocardiography and electrocardiogram were performed and no abnormalities were identified. In the proband, neither cardiomyopathy nor atrioventricular cardiac conduction defects (third-degree) was documented.

At the age of 33 years, the patient was examined by the authors of this study and required the use of a hearing aid. His cognitive abilities remained normal. He obtained a university education and worked in a graduate level position. However, he denied paraesthesia.

**Figure 1:**
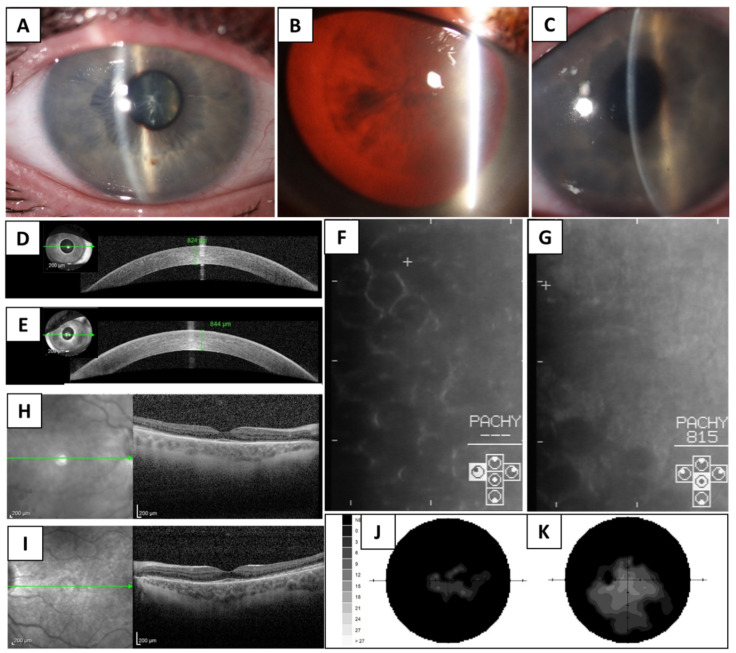
Panocular involvement in a case with Kearns-Sayre syndrome spectrum. Corneal oedema in the right eye (**A**), nuclear cataract in the right eye (**B**), corneal oedema in the left eye (narrow beam) (**C**), SD-OCT imaging of the right (**D**) and left cornea (**E**) documenting increased central corneal thickness). Specular microscopy images showing corneal endothelial surface with beaten metal appearance (quality is decreased due to stromal haze) in the right (**F**) and left eye (**G**), SD-OCT imaging of the macula documenting disintegration of the photoreceptor layer (arrow), in the right (**H**) and left eye (**I**), static perimetry showing visual field loss in the right (**J**) and left (**K**) eye.

The patient complained of a progressive decrease of visual acuity, nyctalopia, and light sensitivity. His BCVA was 0.04 in the right eye and 0.2 in the left eye. There was an external ocular muscle palsy with a marked limitation of up- and down-gaze, while the horizontal movement was nearly normal. Bilateral ptosis was scored as severe. Slit-lamp biomicroscopy and spectral-domain optical coherence tomography (SD-OCT) documented marked corneal stromal oedema with a central corneal thickness of 824 µm in the right eye and 844 µm in the left eye (Figure 1A,C–E) (normal values ≤ 602 µm) [17]. Specular microscopy revealed the presence of rounded dark cells that resemble cornea guttata (Figure 1F,G). In addition, a nuclear cataract in the right eye (Figure 1B) and artificial lens in the left eye were present.

Retinal pathology, primary at the photoreceptor level, was documented by SD-OCT (Figure 1H,I). Other fundus imaging was not possible due to the anterior segment findings. However, when dilated, bilateral peripheral pigmentary changes (course clumping) were noted upon a slit-lamp examination. A static perimetry technique showed concentric visual field restriction with a reduction in the sensitivity of the central island (Figure 1J,K).

Two years later, a follow-up ocular examination documented further deterioration of visual fields. Corneal oedema remained bilaterally stable when the central corneal thickness was measured by SD-OCT. The possible benefit of lamellar keratoplasty and/or cataract removal in the right eye was considered. However, due to retinopathy contributing to the BCVA decrease together with the patient’s preference, it has been postponed until further progression of corneal oedema or when the cataract is clearly documented.

Exome sequencing successfully captured 96.85% of the coding regions with a minimal coverage of 20×. No possible pathogenic variants in the genes, which are associated with congenital cataracts or corneal dystrophies, were detected.

The CTG18.1 repeat length was determined to be 12 and 33, on each respective allele. A repeat length ≥50 is generally considered to be the cause of Fuchs endothelial corneal dystrophy, as demonstrated by most of the studies [8]. However, a threshold of 31 repeats, previously found to be critical for the occurrence of toxic RNA foci in corneal endothelial cells, was met [18].

The systemic findings, methodology, and results of the mtDNA analysis in the proband have formed part of a recent report that summarizes the clinical data in 20 Czech patients with KSS spectrum. However, an individual case presentation including detailed ocular findings has not been reported [10].

## 4. Discussion

Mitochondrial diseases involve tissues with high-energy requirements. The most common ocular manifestations of KSS include ptosis, progressive chronic external ophthalmoplegia, and pigmentary degeneration of the retina [1,2].

Herein, we describe a 33-year-old male with KSS spectrum due to ~6.5 kb mtDNA deletion, who was also manifested with bilateral corneal oedema and paediatric cataracts. Due to the high metabolic demand of corneal endothelial cells that pump ions for the maintenance of stromal deturgescence [19], one would expect that corneal endothelium affects patients with mitochondrial disease. However, to date, less than 10 patients with the KSS spectrum and marked corneal oedema have been documented [5,6,20]. Regarding paediatric cataract, this is the first report of co-occurrence with KSS. Therefore, it is unclear if the cataract can be considered as an ultrarare sign of this disease or a concurrent condition, especially given the family history of premature cataracts in the diseased mother.

Nevertheless, in this study, we also considered the possibility that the congenital cataracts and/or corneal endothelial dysfunction observed in the proband could be attributed to genetic causes beyond the confirmed mtDNA deletion. On this basis, exome sequencing together with targeted Sanger sequencing did not detect any variants that could be considered as likely pathogenic. The STR analysis revealed that the length of the CTG18.1 repeat expansion on one allele was 33. This length is considered to be borderline with respect to the FECD-associations [18]. In combination with the previous cataract surgery, this repeat length may explain the presentation of corneal oedema in the proband. However, it should be noted that the right eye with the same extent of corneal oedema did not undergo any surgery. Herein, it remains plausible that the CTG18.1 repeat length of 33, in combination with mtDNA deletion harbored by the proband, may have resulted in a cumulatively detrimental effect on the corneal endothelium.

## 5. Conclusions

The exclusion of the most common genetic causes implicated in corneal endothelial dystrophies, further support corneal oedema as a clinical manifestation of mitochondriopathy rather than a separate entity. The role of *TCF4* CTG18.1 should be further clarified. Furthermore, we recommend CTG18.1 genotyping for all cases of mitochondriopathies with corneal endothelial involvement, as these patients may have a lower repeat-length threshold for endothelial failure.

## References

[1] Rowland L.P. (1983). Molecular genetics, pseudogenetics, and clinical neurology. The Robert Wartenberg Lecture. Neurology.

[2] Mancuso M., Orsucci D., Angelini C., Bertini E., Carelli V., Comi G.P., Donati M.A., Federico A., Minetti C., Moggio M. (2015). Redefining phenotypes associated with mitochondrial DNA single deletion. J. Neurol..

[3] Finsterer J., Zarrouk-Mahjoub S. (2016). Corneal Involvement in Kearns-Sayre Syndrome Responsive to Coenzyme-Q?. Cornea.

[4] Ortiz A., Arias J., Cardenas P., Villamil J., Peralta M., Escaf L.C., Ortiz J. (2017). Macular findings in Spectral Domain Optical Coherence Tomography and OCT Angiography in a patient with Kearns-Sayre syndrome. Int. J. Retin. Vitr..

[5] Kim J., Medsinge A., Chauhan B., Wiest C., Scanga H., Monaghan R., Moore W.H., Nischal K.K. (2016). Coenzyme Q10 in the Treatment of Corneal Edema in Kearns-Sayre: Is There an Application in Fuchs Endothelial Corneal Dystrophy?. Cornea.

[6] Gonnermann J., Klamann M.K., Maier A.K., Bertelmann E., Schroeter J., von Au K., Joussen A.M., Torun N. (2014). Descemet membrane endothelial keratoplasty in a child with corneal endothelial dysfunction in Kearns-Sayre syndrome. Cornea.

[7] Evans C.J., Liskova P., Dudakova L., Hrabcikova P., Horinek A., Jirsova K., Filipec M., Hardcastle A.J., Davidson A.E., Tuft S.J. (2015). Identification of six novel mutations in *ZEB1* and description of the associated phenotypes in patients with posterior polymorphous corneal dystrophy 3. Ann. Hum. Genet..

[8] Fautsch M.P., Wieben E.D., Baratz K.H., Bhattacharyya N., Sadan A.N., Hafford-Tear N.J., Tuft S.J., Davidson A.E. (2021). TCF4-mediated Fuchs endothelial corneal dystrophy: Insights into a common trinucleotide repeat-associated disease. Prog. Retin. Eye Res..

[9] Bene J., Nadasi E., Kosztolanyi G., Mehes K., Melegh B. (2003). Congenital cataract as the first symptom of a neuromuscular disease caused by a novel single large-scale mitochondrial DNA deletion. Eur. J. Hum. Genet..

[10] Anteneova N., Kelifova S., Kolarova H., Vondrackova A., Tothova I., Liskova P., Magner M., Zamecnik J., Hansikova H., Zeman J. (2020). The Phenotypic Spectrum of 47 Czech Patients with Single, Large-Scale Mitochondrial DNA Deletions. Brain Sci..

[11] Dudakova L., Evans C.J., Pontikos N., Hafford-Tear N.J., Malinka F., Skalicka P., Horinek A., Munier F.L., Voide N., Studeny P. (2019). The utility of massively parallel sequencing for posterior polymorphous corneal dystrophy type 3 molecular diagnosis. Exp. Eye Res..

[12] Karczewski K.J., Francioli L.C., Tiao G., Cummings B.B., Alfoldi J., Wang Q., Collins R.L., Laricchia K.M., Ganna A., Birnbaum D.P. (2020). The mutational constraint spectrum quantified from variation in 141,456 humans. Nature.

[13] Martin A.R., Williams E., Foulger R.E., Leigh S., Daugherty L.C., Niblock O., Leong I.U.S., Smith K.R., Gerasimenko O., Haraldsdottir E. (2019). PanelApp crowdsources expert knowledge to establish consensus diagnostic gene panels. Nat. Genet..

[14] Liskova P., Dudakova L., Evans C.J., Rojas Lopez K.E., Pontikos N., Athanasiou D., Jama H., Sach J., Skalicka P., Stranecky V. (2018). Ectopic *GRHL2* Expression Due to Non-coding Mutations Promotes Cell State Transition and Causes Posterior Polymorphous Corneal Dystrophy 4. Am. J. Hum. Genet..

[15] Davidson A.E., Liskova P., Evans C.J., Dudakova L., Noskova L., Pontikos N., Hartmannova H., Hodanova K., Stranecky V., Kozmik Z. (2016). Autosomal-Dominant Corneal Endothelial Dystrophies CHED1 and PPCD1 Are Allelic Disorders Caused by Non-coding Mutations in the Promoter of *OVOL2*. Am. J. Hum. Genet..

[16] Wieben E.D., Aleff R.A., Tosakulwong N., Butz M.L., Highsmith W.E., Edwards A.O., Baratz K.H. (2012). A common trinucleotide repeat expansion within the transcription factor 4 (TCF4, E2-2) gene predicts Fuchs corneal dystrophy. PLoS ONE.

[17] Gilani F., Cortese M., Ambrosio R.R., Lopes B., Ramos I., Harvey E.M., Belin M.W. (2013). Comprehensive anterior segment normal values generated by rotating Scheimpflug tomography. J. Cataract Refract. Surg..

[18] Zarouchlioti C., Sanchez-Pintado B., Hafford Tear N.J., Klein P., Liskova P., Dulla K., Semo M., Vugler A.A., Muthusamy K., Dudakova L. (2018). Antisense Therapy for a Common Corneal Dystrophy Ameliorates TCF4 Repeat Expansion-Mediated Toxicity. Am. J. Hum. Genet..

[19] Bourne W.M. (2003). Biology of the corneal endothelium in health and disease. Eye.

[20] Chang T.S., Johns D.R., Stark W.J., Drachman D.B., Green W.R. (1994). Corneal decompensation in mitochondrial ophthalmoplegia plus (Kearns-Sayre) syndrome. A clinicopathologic case report. Cornea.

